# Clinical models to predict lymph nodes metastasis and distant metastasis in newly diagnosed early esophageal cancer patients: A population‐based study

**DOI:** 10.1002/cam4.5334

**Published:** 2022-10-07

**Authors:** Hong Chen, Junxian Wu, Wanting Guo, Lihang Yang, Linbin Lu, Yihong Lin, Xuewen Wang, Yan Zhang, Xi Chen

**Affiliations:** ^1^ Department of Oncology, The 900th Hospital of the People's Liberation Army Joint Service Support Force Fuzong Clinical Medical College of Fujian Medical University Fuzhou Fujian 350025 China; ^2^ Department of Gastroenterology Quanzhou First Hospital Affiliated to Fujian Medical University Quanzhou Fujian 362000 China

**Keywords:** distant metastasis, lymph nodes metastasis, nomogram, SEER, T1 esophageal cancer

## Abstract

**Background:**

Patients with early esophageal cancer (EC) receive individualized therapy based on their lymph node metastasis (LNM) and distant metastasis (DM) status; however, deficiencies in current clinical staging techniques and the issue of cost‐effectiveness mean LNM and DM often go undetected preoperatively. We aimed to develop three clinical models to predict the likelihood of LNM, DM, and prognosis in patients with early EC.

**Method:**

The Surveillance, Epidemiology, and End Results database was queried for T1 EC patients from 2004 to 2015. Multivariable logistic regression and Cox proportional hazards models were used to recognize the risk factors of LNM and DM, predict overall survival (OS), and develop relevant nomograms. Receiver operating characteristic (ROC)/concordance index and calibration curves were used to evaluate the discrimination and accuracy of the three nomograms. Decision curve analyses (DCAs), clinical impact curves, and subgroups based on model scores were used to determine clinical practicability.

**Results:**

The area under the curve of the LNM and DM nomograms were 0.668 and 0.807, respectively. The corresponding C‐index of OS nomogram was 0.752. Calibration curves and DCA showed an effective predictive accuracy and clinical applicability. In patients with T1N0M0 EC, surgery alone (*p* < 0.01) proved a survival advantage. Chemotherapy and radiotherapy indicated a better prognosis in the subgroup analysis for T1 EC patients with LNM or DM.

**Conclusions:**

We created three nomograms to predict the likelihood of LNM, DM, and OS probability in patients with early EC using a generalizable dataset. These useful visual tools could help clinical physicians deliver appropriate perioperative care.

## Graphical abstract

Deficiencies in current clinical staging techniques and the issue of costeffectiveness mean lymph nodes metastasis and distant metastasis often go undetected preoperatively in early esophageal cancer. We developed and validated three nomograms to predict the likelihood of lymph nodes metastasis, distant metastasis, and overall survival.

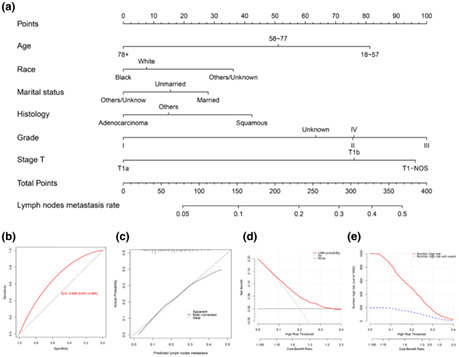

## 1 INTRODUCTION

Esophageal cancer (EC) is the seventh most common tumor, with 604,000 new cases and 544,000 deaths in 2020 worldwide.^1^ Early EC is defined as EC limited to the mucosa (T1a) or submucosa (T1b); regardless of lymph node status,^2^ it accounts for 20% of initially diagnosed cases.^3^ However, the incidence of lymph node metastasis (LNM) is still high in early EC, especially for tumors with submucosal infiltration.^4^, ^5^ In a prospective study from Japan, LNM occurred in 27% of patients with early EC, as determined by pathological examinations.^6^ The occurrence of LNM and distant metastasis (DM) suggests a poor prognosis in patients with EC, especially in early‐stage disease. Thus, the LNM and DM status need to be considered in the treatment options and timing of patients with early EC.

Radical esophagectomy and lymph node dissection have been the standard treatment for early‐ and advanced‐EC. However, the incidence of complications and the postoperative mortality are still high, seriously affecting the quality of life in patients with EC who undergo esophagectomy. To reduce the probability of postoperative complications and mortality, endoscopic therapy has increasingly become an alternative treatment modality for patients with early EC.^7^ Some previously published studies have demonstrated that the prognosis of patients with early EC treated with radical surgery or endoscopic resection was equivalent^8^, ^9^, ^10^; for the patients with LNM, however, endoscopic resection can lead to disease recurrence and poor prognosis, given that a cure is sometimes not achieved. Endoscopic ultrasound (EUS) is typically performed to assess the T stage of patients with EC, with high accuracy; however, this procedure has limitations in discovering occult nodal metastases.^11^, ^12^ Current staging systems are inadequate for appropriate risk stratification of patients with early EC. Therefore, it is of great value to develop a clinical tool to predict LNM and DM in early EC, given that it has significant clinical importance and could be helpful for developing an optimal treatment regimen.

Thus, we have developed clinical risk prediction models of early EC based on the Surveillance, Epidemiology, and End Results (SEER) database. We aim to quantify individual risk based on clinical data from patients with early EC. For patients with a high‐risk of LNM, esophagectomy might be more appropriate. For those with low‐risk of LNM, endoscopic therapy would be better, given that it can spare the additional risks and costs of radical surgery. For those with high mortality risk, however, intensive treatment is necessary. In our study, we aim to (1) recognize the independent risk factors for LNM and DM in early EC patients, then establish the corresponding nomograms; (2) conduct survival analyses and develop an OS nomogram; (3) analyze the influence of various treatments on prognosis in early EC patients.

## 2 METHODS

### 2.1 Patients

The records of the patients were extracted from the SEER program, the United States authoritative cancer statistics database, accounting for approximately 35% of the US population. SEER*Stat (version 8.3.9.2) was used to screen eligible patients in our study. No ethical approval was required for our study because the SEER database was publicly available as open‐access data.

**FIGURE 1:**
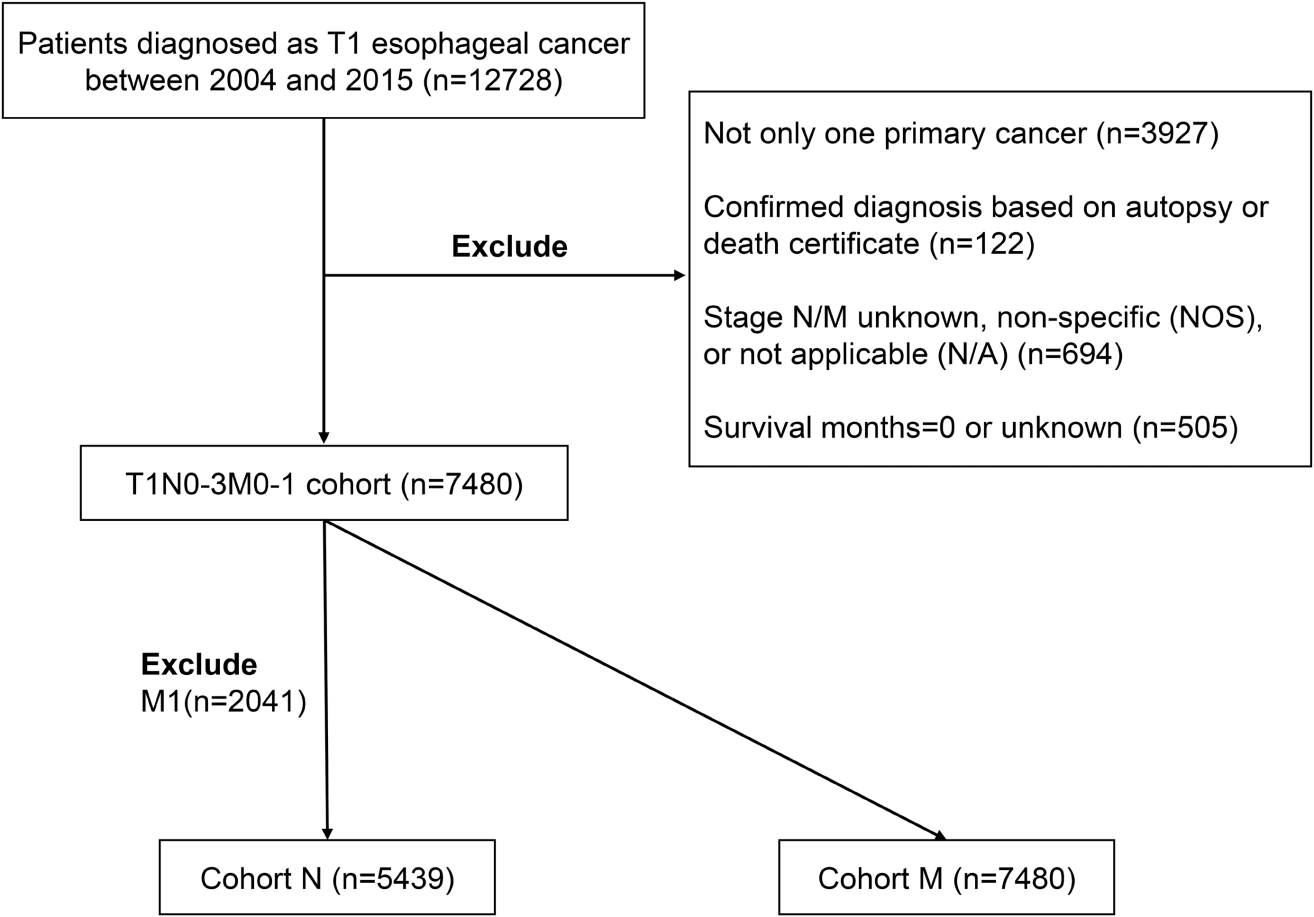
Research flowchart

7480 patients diagnosed with T1 EC were enrolled within the SEER database from 2004 to 2015. The exclusion criteria were as follows: (1) patients whose EC was not the primary cancer; (2) confirmed diagnosis based on autopsy or death certificate; (3) N/M stage was non‐specific (NOS), not applicable (N/A), or unknown; and (4) survival months were 0 or unknown. The flow chart of case selection is represented in Figure 1. Based on existing evidence‐based medicine, the LNM would not determine the treatment of patients with distant metastatic disease; however, it could change treatment decisions in patients without distant metastatic disease. Thus, we divided all patients into two groups: cohort N (*n* = 5439), comprising patients with T1N0‐3 M0 EC, was set to predict LNM; cohort M (*n* = 7480), formed of the T1N0‐3 M0‐1 EC population, was set to predict DM.

### 2.2 Variable declaration and outcomes

The variables selected from the SEER database were as follows: year of diagnosis, age at diagnosis, race, sex, insurance status, marital status, histology, primary site, grade, TNM stage, surgery record, chemotherapy record, radiation record, survival months, vital status, and cause of death.

Variables mentioned above were divided into three distinct categories: demographic, tumor‐related, and treatment‐related. For the demographic variables, we subclassified the patients by age at diagnosis (18–57, 58–77, 78+), year of diagnosis (2004–2007, 2008–2011, 2012–2015), insurance status (insured, uninsured/unknown), race (White, Black, other/unknown), and marital status (married, unmarried, other/unknown). The tumor‐related covariates were according to histological differentiation (Grade I, Grade II, Grade III, Grade IV, unknown). The primary site was reclassified based on the International Classification of Diseases for Oncology (ICD‐O‐2) codes (upper: C15.0 and C15.3; middle: C15.4 and C15.1; lower: C15.2 and C15.5; other: overlapping lesions/NOS). Histology was defined by the International Classification of Diseases for Oncology, 3rd Edition (ICD‐O‐3) (adenocarcinoma: 8140–8389, 8480, 8481, 8570, 8574; squamous cell carcinoma: 8050–8089; other), and the TNM stage was reclassified according to the AJCC 8th edition staging system. Treatment‐related variables included surgery (no/unknown, yes), radiation (no/unknown, yes), and chemotherapy (no/unknown, yes).

The primary endpoint was overall survival (OS), defined as the duration from diagnosis to mortality or the last follow‐up. We established cancer‐specific survival (CSS) as the secondary endpoint, defined as the time interval between diagnosis and death from EC.

### 2.3 Nomogram construction and validation

Multivariable logistic regression and Cox proportional hazards models were used to identify LNM, DM risk factors, and predict OS. Variables associated with LNM and DM were selected to derive a nomogram for LNM and DM for T1 EC. Variables included in the multivariate Cox regression model were based on both statistical significance and their clinical significance, which were selected to develop a prognostic nomogram. We drew receiver operating characteristic (ROC) curves and calculated the area under the curves (AUC) to assess the discrimination of the three nomograms. Calibration curves were conducted using a bootstrapping method were plotted to assess the calibration. A decision curve analysis (DCA) was used to assess the clinical utility. OS was evaluated using the Kaplan–Meier (KM) method, and CSS was determined by cumulative incidence function. Clinical impact curves (CICs) were performed to reveal the significance value of the nomograms. All patients were divided into three groups based on the quartiles of the nomogram scores to assess the clinical utility of the nomograms.

### 2.4 Statistical analysis

All the statistical analyses were undertaken using R software (version 4.1.1). Clinicopathological characteristics were assessed using Student's t‐test and chi‐squared test. The nomograms, ROC, calibration, DCA, clinical impact curve, and Kaplan–Meier curves were plotted by R packages and functions. A two‐sided *p* < 0.05 was considered statistically significant.

## 3 RESULTS

### 3.1 Clinicopathologic characteristics of patients with T1 esophageal cancer

Based on the inclusion criteria, 7480 patients diagnosed with T1 EC were included from the SEER database for the final analysis. We divided the study population into two groups according to LNM and DM status: cohort N (T1N0‐3 M0 EC, *n* = 5439) and cohort M (T1N0‐3 M0‐1 EC, *n* = 7480). The incidence of LNM in cohort N was 20.04%; DM occurred in 2041 patients in cohort M, at a rate of 27.29%. The clinicopathological parameters of T1 EC patients are shown in Table 1.

**TABLE 1:** Clinicopathological variables of patients with T1 esophageal cancer

Characteristic	Nt (%)	Nne (%)	Ne (%)	*P*	Mt (%)	Mne (%)	Me (%)	*p*
	*N* = 5439	*N* = 4349	*N* = 1090		*N* = 7480	*N* = 5439	*N* = 2041	
Year of diagnosis								
2004–2007	1861 (34.2)	1509 (34.7)	352 (32.3)	0.27	2485 (33.2)	1861 (34.2)	624 (30.6)	0.01
2008–2011	1841 (33.8)	1469 (33.8)	372 (34.1)		2566 (34.3)	1841 (33.8)	725 (35.5)	
2012–2015	1737 (31.9)	1371 (31.5)	366 (33.6)		2429 (32.5)	1737 (31.9)	692 (33.9)	
Age at diagnosis								
18 ~ 57	1068 (19.6)	805 (18.5)	263 (24.1)	<0.01	1675 (22.4)	1068 (19.6)	607 (29.7)	<0.01
57 ~ 77	3145 (57.8)	2497 (57.4)	648 (59.4)		4301 (57.5)	3145 (57.8)	1156 (56.6)	
78+	1226 (22.5)	1047 (24.1)	179 (16.4)		1504 (20.1)	1226 (22.5)	278 (13.6)	
Gender								
Male	4222 (77.6)	3361 (77.3)	861 (79.0)	0.24	5895 (78.8)	4222 (77.6)	1673 (82.0)	<0.01
Female	1217 (22.4)	988 (22.7)	229 (21.0)		1585 (21.2)	1217 (22.4)	368 (18.0)	
Race								
White	4589 (84.4)	3717 (85.5)	872 (80.0)	<0.01	6317 (84.5)	4589 (84.4)	1728 (84.7)	0.95
Black	553 (10.2)	419 (9.6)	134 (12.3)		756 (10.1)	553 (10.2)	203 (9.9)	
Others/Unknown	297 (5.5)	213 (4.9)	84 (7.7)		407 (5.4)	297 (5.5)	110 (5.4)	
Insurance status								
Insured	3863 (71.0)	3064 (70.5)	799 (73.3)	0.07	5372 (71.8)	3863 (71.0)	1509 (73.9)	0.01
Uninsured/Unknown	1576 (29.0)	1285 (29.5)	291 (26.7)		2108 (28.2)	1576 (29.0)	532 (26.1)	
Marital status								
Married	2977 (54.7)	2354 (54.1)	623 (57.2)	0.01	4123 (55.1)	2977 (54.7)	1146 (56.1)	<0.01
Unmarried	833 (15.3)	652 (15.0)	181 (16.6)		1200 (16.0)	833 (15.3)	367 (18.0)	
Others/Unknown	1629 (30.0)	1343 (30.9)	286 (26.2)		2157 (28.8)	1629 (30.0)	528 (25.9)	
Primary site								
Upper	352 (6.5)	281 (6.5)	71 (6.5)	<0.01	435 (5.8)	352 (6.5)	83 (4.1)	<0.01
Middle/Thoracic	1107 (20.4)	835 (19.2)	272 (25.0)		1456 (19.5)	1107 (20.4)	349 (17.1)	
Lower	3322 (61.1)	2685 (61.7)	637 (58.4)		4686 (62.6)	3322 (61.1)	1364 (66.8)	
Others/Unknown	658 (12.1)	548 (12.6)	110 (10.1)		903 (12.1)	658 (12.1)	245 (12.0)	
Histology type								
Adenocarcinoma	3452 (63.5)	2868 (65.9)	584 (53.6)	<0.01	4755 (63.6)	3452 (63.5)	1303 (63.8)	<0.01
Squamous	1623 (29.8)	1193 (27.4)	430 (39.4)		2179 (29.1)	1623 (29.8)	556 (27.2)	
Others	364 (6.7)	288 (6.6)	76 (7.0)		546 (7.3)	364 (6.7)	182 (8.9)	
Pathology grade								
Grade I	477 (8.8)	433 (10.0)	44 (4.0)	<0.01	531 (7.1)	477 (8.8)	54 (2.6)	<0.01
Grade II	1983 (36.5)	1581 (36.4)	402 (36.9)		2571 (34.4)	1983 (36.5)	588 (28.8)	
Grade III	1638 (30.1)	1218 (28.0)	420 (38.5)		2666 (35.6)	1638 (30.1)	1028 (50.4)	
Grade IV	83 (1.5)	66 (1.5)	17 (1.6)		125 (1.7)	83 (1.5)	42 (2.1)	
Unknown	1258 (23.1)	1051 (24.2)	207 (19.0)		1587 (21.2)	1258 (23.1)	329 (16.1)	
T staging								
T1a	1812 (33.3)	1621 (37.3)	191 (17.5)	<0.01	2098 (28.0)	1812 (33.3)	286 (14.0)	<0.01
T1b	1029 (18.9)	798 (18.3)	231 (21.2)		1121 (15.0)	1029 (18.9)	92 (4.5)	
T1‐NOS	2598 (47.8)	1930 (44.4)	668 (61.3)		4261 (57.0)	2598 (47.8)	1663 (81.5)	
N staging								
N0	NA	NA	NA	NA	5134 (68.6)	4349 (80.0)	785 (38.5)	<0.01
N1	NA	NA	NA		1350 (18.0)	647 (11.9)	703 (34.4)	
N2	NA	NA	NA		146 (2.0)	80 (1.5)	66 (3.2)	
N3	NA	NA	NA		58 (0.8)	26 (0.5)	32 (1.6)	
N1‐NOS	NA	NA	NA		792 (10.6)	337 (6.2)	455 (22.3)	
Surgery								
No/Unknown	3025 (55.6)	2281 (52.4)	744 (68.3)	<0.01	4995 (66.8)	3025 (55.6)	1970 (96.5)	<0.01
Yes	2414 (44.4)	2068 (47.6)	346 (31.7)		2485 (33.2)	2414 (44.4)	71 (3.5)	
Radiation								
No/Unknown	3081 (56.6)	2755 (63.3)	326 (29.9)	<0.01	4206 (56.2)	3081 (56.6)	1125 (55.1)	0.25
Yes	2358 (43.4)	1594 (36.7)	764 (70.1)		3274 (43.8)	2358 (43.4)	916 (44.9)	
Chemotherapy								
No/Unknown	3190 (58.7)	2909 (66.9)	281 (25.8)	<0.01	3862 (51.6)	3190 (58.7)	672 (32.9)	<0.01
Yes	2249 (41.3)	1440 (33.1)	809 (74.2)		3618 (48.4)	2249 (41.3)	1369 (67.1)	
Survival status								
Alive	1907 (35.1)	1671 (38.4)	236 (21.7)	<0.01	2001 (26.8)	1907 (35.1)	94 (4.6)	<0.01
Dead of cancer	2715 (49.9)	1990 (45.8)	725 (66.5)		4570 (61.1)	2715 (49.9)	1855 (90.9)	
Dead of other cause	817 (15.0)	688 (15.8)	129 (11.8)		909 (12.2)	817 (15.0)	92 (4.5)	
Survival months (mean ± sd)	36.00 ± 37.51	38.55 ± 38.85	25.842 ± 29.53	<0.01	28.99 ± 34.83	36.00 ± 37.51	10.31 ± 14.67	<0.01

*Note*: Nt (%), total number of the cohort N; Mt (%), total number of the cohort M; Ne (%), number of LNM events; Me (%), number of DM events; Nne (%), number of non‐LNM events; Mne (%), number of non‐DM events.

Abbreviations: DM, distant metastasis; LNM, lymph node metastasis; NOS, not otherwise specified.

### 3.2 Predictors of lymph node metastasis and construction of the nomogram

Based on the univariable and multivariable logistic regression analyses, six variables were considered as independent risk factors to predict LNM in cohort N as follows (Table 2): age at diagnosis, marital status, race, histology type, T stage, and histologic differentiation. Older patients had a lower risk of LNM, especially for those aged over 78 years (odds ratio [OR] 0.44, 95% CI 0.35–0.55, *p* < 0.01). Compared with Whites, all the other races (excluding Black) had a higher risk of LNM (OR 1.34, 95% CI 1.01–1.77, *p* = 0.04). The patients with other/unknown marital status had a lower risk of LNM (OR 0.75, 95% CI 0.64–0.89, *p* < 0.01). In terms of histology type, a higher risk of LNM was found in patients with squamous cell EC (OR 1.54, 95% CI 1.3–1.82, *p* < 0.01). T1 EC patients with moderately differentiated (OR 2.16, 95% CI 1.54–3.02, *p* < 0.01), poorly differentiated (OR 2.77, 95% CI 1.97–3.87, *p* < 0.01), or undifferentiated (OR 2.17, 95% CI 1.15–4.09, *p* = 0.017) all had a higher risk of LNM. As for T stage, T1b (OR 2.17, 95% CI 1.75–2.69, *p* < 0.01) and T1‐NOS (OR 2.66, 95% CI 2.22–3.19, *p* < 0.01) EC were more likely to have LNM.

**TABLE 2:** Logistic regression analysis of variables associated with LNM in T1N0‐3 M0 esophageal cancer

Variables	Univariate analysis		Multivariate analysis	
	OR (95% CI)	*p*	OR (95% CI)	*p*
Year of diagnosis				
2004–2007	Reference			
2008–2011	1.09 (0.92–1.28)	0.32		
2012–2015	1.14 (0.97–1.35)	0.11		
Age at diagnosis				
18 ~ 57	Reference		Reference	
58 ~ 77	0.79 (0.67–0.94)	<0.01	0.74 (0.62–0.87)	<0.01
78+	0.52 (0.42–0.65)	<0.01	0.44 (0.35–0.55)	<0.01
Gender				
Male	Reference			
Female	0.9 (0.77–1.06)	0.23		
Race				
White	Reference		Reference	
Black	1.36 (1.11–1.68)	<0.01	0.92 (0.73–1.17)	0.52
Others/Unknown	1.68 (1.29–2.19)	<0.01	1.34 (1.01–1.77)	0.04
Insurance status				
Insured	Reference			
Uninsured	0.87 (0.75–1.01)	0.06		
Marital status				
Married	Reference		Reference	
Unmarried	1.05 (0.87–1.26)	0.62	0.88 (0.72–1.07)	0.21
Others/Unknown	0.8 (0.69–0.94)	<0.01	0.75 (0.64–0.89)	<0.01
Primary site				
Upper	Reference			
Middle/Thoracic	1.29 (0.96–1.73)	0.09		
Lower	0.94 (0.71–1.24)	0.65		
Others/Unknown	0.79 (0.57–1.11)	0.17		
Histology type				
Adenocarcinoma	Reference		Reference	
Squamous	1.77 (1.54–2.04)	<0.01	1.54 (1.3–1.82)	<0.01
Others	1.3 (0.99–1.69)	0.06	1.16 (0.88–1.55)	0.29
Pathology grade				
Grade I	Reference		Reference	
Grade II	2.5 (1.8–3.48)	<0.01	2.16 (1.54–3.02)	<0.01
Grade III	3.39 (2.44–4.72)	<0.01	2.77 (1.97–3.87)	<0.01
Grade IV	2.53 (1.37–4.7)	<0.01	2.17 (1.15–4.09)	0.02
Unknown	1.94 (1.37–2.73)	<0.01	1.91 (1.34–2.71)	<0.01
T staging				
T1a	Reference		Reference	
T1b	2.46 (1.99–3.03)	<0.01	2.17 (1.75–2.69)	<0.01
T1‐NOS	2.94 (2.47–3.5)	<0.01	2.66 (2.22–3.19)	<0.01

Abbreviations: 95% CI, 95% confidence intervals; LNM, lymph node metastasis; NOS, not otherwise specified; OR, odd ratio.

**FIGURE 2:**
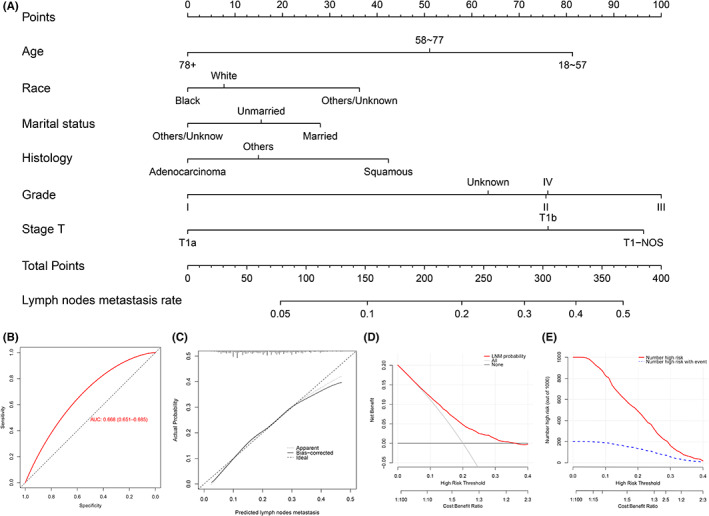
A nomogram (A) for forecasting LNM in cohort N. ROC curve (B), calibration curve (C), DCA curve (D), and the relevant CIC (E) of the LNM nomogram

To illustrate the LNM risk in cohort N more intuitively, we established a nomogram to visualize the risk model (Figure 2A). Histological differentiation accounted for the maximum portion in the nomogram, followed by T stage, age, histology type, race, and marital status. The ROC (Figure 2B) displayed good discrimination with an AUC of 0.668 (95% CI 0.651–0.685). Meanwhile, an effective accuracy was presented by the calibration curve (Figure 2C). The DCA (Figure 2D) for prediction of LNM in TI EC showed the highest net benefit across 0%–40% threshold probabilities of the nomogram. CICs (Figure 2E) were plotted to assess the clinical applicability of this nomogram.

### 3.3 Independent risk factors of distant metastasis and nomogram

We conducted a univariable and multivariable logistic regression analysis to identify the independent risk variables for DM. Six independent risk factors were identified (Table 3): age at diagnosis, primary site, histology type, grade, T stage, and N stage. A decreasing LNM occurrence was found in the elderly (age 58–77, OR 0.66, 95% CI 0.57–0.76, *p* < 0.01; age 78 and older, OR 0.39, 95% CI 0.32–0.48, *p* < 0.01). In terms of the primary site, T1 EC patients with a lower site (OR 1.66, 95% CI 1.23–2.23, *P* < 0.01) were more likely to present with DM. Compared with patients with T1 esophageal adenocarcinoma, those with squamous cell EC (OR 0.74, 95% CI 0.63–0.87, *p* < 0.01) were at a lower risk of DM. DM frequently occurred in T1 EC with poorer differentiation: Grade II (OR 1.88, 95% CI 1.36–2.61, *p* < 0.01), Grade III (OR 3.36, 95% CI 2.43–4.65, *p* < 0.01), and Grade IV (OR 2.92, 95% CI 1.71–4.98, *p* < 0.01). We also found that tumors with an invasion depth of T1b (OR 0.39, 95% CI 0.3–0.51, *p* < 0.01) were at a lower risk of DM, whereas T1‐NOS (OR 3.3, 95% CI 2.83–3.85, *p* < 0.01) tumors were at higher risk of DM. T1 EC patients with LNM were at a higher risk of DM (N1, OR 4.76, 95% CI 4.1–5.52, *p* < 0.01; N2, OR 3.93, 95% CI 2.71–5.69, *p* < 0.01; N3, OR 5.07, 95% CI 2.83–9.06, *p* < 0.01; N1‐NOS, OR 5.77, 95% CI 4.83–6.89, *p* < 0.01) compared with lymph node‐negative patients.

**TABLE 3:** Logistic regression analysis of the risk factors for DM in T1 T1N0‐3 M0‐1 esophageal cancer

Variables	Univariate analysis		Multivariate analysis	
	OR (95% CI)	*p*	OR (95% CI)	*p*
Year of diagnosis				
2004–2007	Reference		Reference	
2008–2011	1.17 (1.04–1.33)	<0.01	1.07 (0.88–1.3)	0.50
2012–2015	1.19 (1.05–1.35)	<0.01	1.14 (0.92–1.4)	0.22
Age at diagnosis				
18 ~ 57	Reference			
58 ~ 77	0.65 (0.57–0.73)	<0.01	0.66 (0.57–0.76)	<0.01
78+	0.4 (0.34–0.47)	<0.01	0.39 (0.32–0.48)	<0.01
Gender				
Male	Reference		Reference	
Female	0.76 (0.67–0.87)	<0.01	0.95 (0.81–1.11)	0.50
Race				
White	Reference			
Black	0.97 (0.82–1.16)	0.77		
Others/Unknown	0.98 (0.78–1.23)	0.89		
Insurance status				
Insured	Reference		Reference	
Uninsured	0.86 (0.77–0.97)	<0.01	0.86 (0.71–1.05)	0.13
Marital status				
Married	Reference		Reference	
Unmarried	1.14 (0.99–1.32)	0.06	0.98 (0.83–1.16)	0.86
Others/Unknown	0.84 (0.75–0.95)	<0.01	0.89 (0.78–1.03)	0.19
Primary site				
Upper	Reference		Reference	
Middle/Thoracic	1.34 (1.02–1.75)	0.03	1.18 (0.87–1.59)	0.28
Lower	1.74 (1.36–2.23)	<0.01	1.66 (1.23–2.23)	<0.01
Others/Unknown	1.58 (1.19–2.09)	<0.01	1.59 (1.15–2.2)	<0.01
Histology type				
Adenocarcinoma	Reference		Reference	
Squamous	0.91 (0.81–1.02)	0.1	0.74 (0.63–0.87)	<0.01
Others	1.32 (1.1–1.6)	<0.01	0.9 (0.72–1.13)	0.37
Pathology grade				
Grade I	Reference		Reference	
Grade II	2.62 (1.95–3.52)	<0.01	1.88 (1.36–2.61)	<0.01
Grade III	5.54 (4.14–7.42)	<0.01	3.36 (2.43–4.65)	<0.01
Grade IV	4.47 (2.81–7.12)	<0.01	2.92 (1.71–4.98)	<0.01
Unknown	2.31 (1.7–3.14)	<0.01	1.71 (1.22–2.39)	<0.01
Stage T				
T1a	Reference		Reference	
T1b	0.57 (0.44–0.73)	<0.01	0.39 (0.3–0.51)	<0.01
T1‐NOS	4.06 (3.53–4.66)	<0.01	3.3 (2.83–3.85)	<0.01
Stage N				
N0	Reference		Reference	
N1	6.02 (5.28–6.86)	<0.01	4.76 (4.1–5.52)	<0.01
N2	4.57 (3.27–6.39)	<0.01	3.93 (2.71–5.69)	<0.01
N3	6.82 (4.04–11.5)	<0.01	5.07 (2.83–9.06)	<0.01
N1‐NOS	7.48 (6.37–8.78)	<0.01	5.77 (4.83–6.89)	<0.01

Abbreviations: 95% CI, 95% confidence intervals; DM, distant metastasis; NOS, not otherwise specified; OR, odd ratio.

**FIGURE 3:**
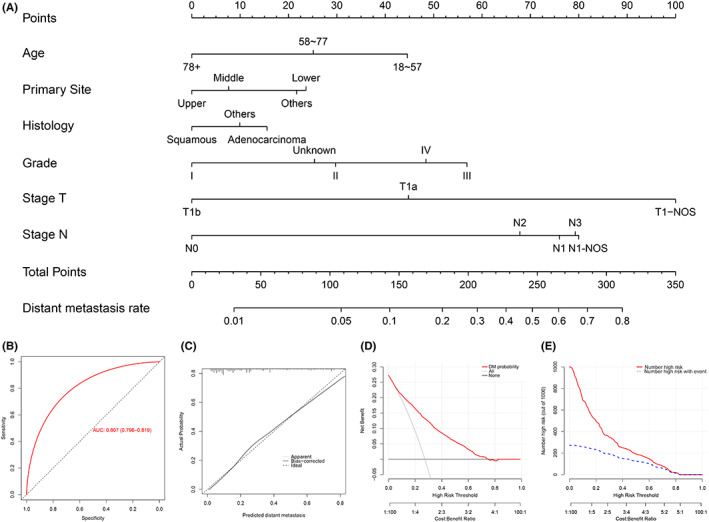
A nomogram (A) for forecasting DM in cohort M. ROC curve (B), calibration curve (C), DCA curve (D), and the relevant CIC (E) of the DM nomogram

Based on the multivariate analysis, a nomogram to predict DM in T1 EC was established. As shown in Figure 3A, the T stage accounted for the largest proportion, followed by the N stage, grade, age at diagnosis, primary site, and histological type. The ROC curve was then plotted to evaluate the efficacy of the proposed model, indicating a nearly perfect AUC (Figure 3B), 0.807 (95% CI 0.796–0.819). The calibration curve is shown by the dashed line in Figure 3C. We observed that the calibration curve nearly coincided with the ideal diagonal line, which showed excellent predictive accuracy. The DCA and CIC (Figure 3D,E) showed that 0–0.8 was the most beneficial threshold probability for predicting DM in this nomogram.

### 3.4 Establishment of prognostic nomogram for patients with T1 esophageal cancer

Based on a univariate Cox proportional hazards regression analysis, we found significant variables, including year, age, sex, race, insurance status, marital status, primary site, grade, histology type, T stage, N stage, M stage, surgery, radiation, and chemotherapy, as shown in Table 4. Four variables were excluded, including year, race, marital status, and insurance status, given that these factors would not be clinically meaningful; the rest were included in the multivariate analysis. Patients with T1 EC and LNM (N1, HR 1.23, 95% CI 1.14–1.32, *p* < 0.01; N3, HR 1.47, 95% CI 1.12–1.93, *p* < 0.01; N1‐NOS, HR 1.14, 95% CI 1.05–1.25, *p* < 0.01) were associated with poor prognosis. A similar result was found in patients with T1 EC and DM (HR 2.05, 95% CI 1.91–2.2, *p* < 0.01).

**TABLE 4:** COX regression analysis of the prognostic factors for overall survival in T1 esophageal cancer

Variables	Univariate analysis		Multivariate analysis	
	HR (95% CI)	*p*	HR (95% CI)	*p*
Year of diagnosis				
2004–2007	Reference			
2008–2011	0.91 (0.85–0.97)	<0.01		
2012–1015	0.81 (0.76–0.87)	<0.01		
Age at diagnosis				
18 ~ 57	Reference		Reference	
58 ~ 77	1.06 (0.99–1.14)	0.08	1.13 (1.05–1.21)	<0.01
78+	1.84 (1.7–2)	<0.01	1.58 (1.45–1.72)	<0.01
Gender				
Male	Reference		Reference	
Female	1.17 (1.1–1.25)	<0.01	0.97 (0.9–1.03)	0.31
Race				
White	Reference			
Black	1.52 (1.4–1.65)	<0.01		
Others/Unknown	1.08 (0.96–1.22)	0.19		
Insurance status				
Insured	Reference			
Uninsured/Unknown	1.18 (1.11–1.25)	<0.01		
Marital status				
Married	Reference			
Unmarried	1.22 (1.13–1.31)	<0.01		
Others/Unknown	1.41 (1.32–1.49)	<0.01		
Primary site				
Upper	Reference		Reference	
Middle/Thoracic	1.04 (0.92–1.18)	0.49	1.17 (1.04–1.33)	<0.01
Lower	0.82 (0.74–0.92)	<0.01	1.21 (1.07–1.37)	<0.01
Others/Unknown	1.02 (0.9–1.16)	0.77	1.24 (1.09–1.42)	<0.01
Histology type				
Adenocarcinoma	Reference		Reference	
Squamous	1.61 (1.52–1.7)	<0.01	1.19 (1.1–1.27)	<0.01
Others	1.48 (1.33–1.63)	<0.01	1.19 (1.07–1.32)	<0.01
Pathology grade				
Grade I	Reference		Reference	
Grade II	1.75 (1.54–1.98)	<0.01	1.17 (1.03–1.33)	0.02
Grade III	2.51 (2.22–2.85)	<0.01	1.39 (1.22–1.58)	<0.01
Grade IV	2.12 (1.68–2.66)	<0.01	1.23 (0.98–1.55)	0.08
Unknown	1.43 (1.25–1.64)	<0.01	0.88 (0.77–1.01)	0.07
Stage T				
T1a	Reference		Reference	
T1b	0.93 (0.84–1.03)	0.15	1.14 (1.03–1.26)	0.01
T1‐NOS	2.9 (2.71–3.1)	<0.01	1.53 (1.42–1.65)	<0.01
Stage N				
N0	Reference		Reference	
N1	1.78 (1.67–1.91)	<0.01	1.23 (1.14–1.32)	<0.01
N2	1.43 (1.18–1.72)	<0.01	1.17 (0.97–1.42)	0.10
N3	2.29 (1.75–3)	<0.01	1.47 (1.12–1.93)	<0.01
N1‐NOS	2.21 (2.04–2.4)	<0.01	1.14 (1.05–1.25)	<0.01
Stage M				
M0	Reference		Reference	
M1	3.32 (3.13–3.51)	<0.01	2.05 (1.91–2.2)	<0.01
Surgery				
No/Unknown	Reference		Reference	
Yes	0.19 (0.17–0.2)	<0.01	0.26 (0.24–0.28)	<0.01
Radiation				
No/Unknown	Reference		Reference	
Yes	1.42 (1.35–1.5)	<0.01	0.99 (0.92–1.05)	0.65
Chemotherapy				
No/Unknown	Reference		Reference	
Yes	1.45 (1.37–1.53)	<0.01	0.64 (0.6–0.68)	<0.01

Abbreviations: 95% CI, 95% confidence intervals; HR, hazard ratio; NOS, not otherwise specified.

**FIGURE 4:**
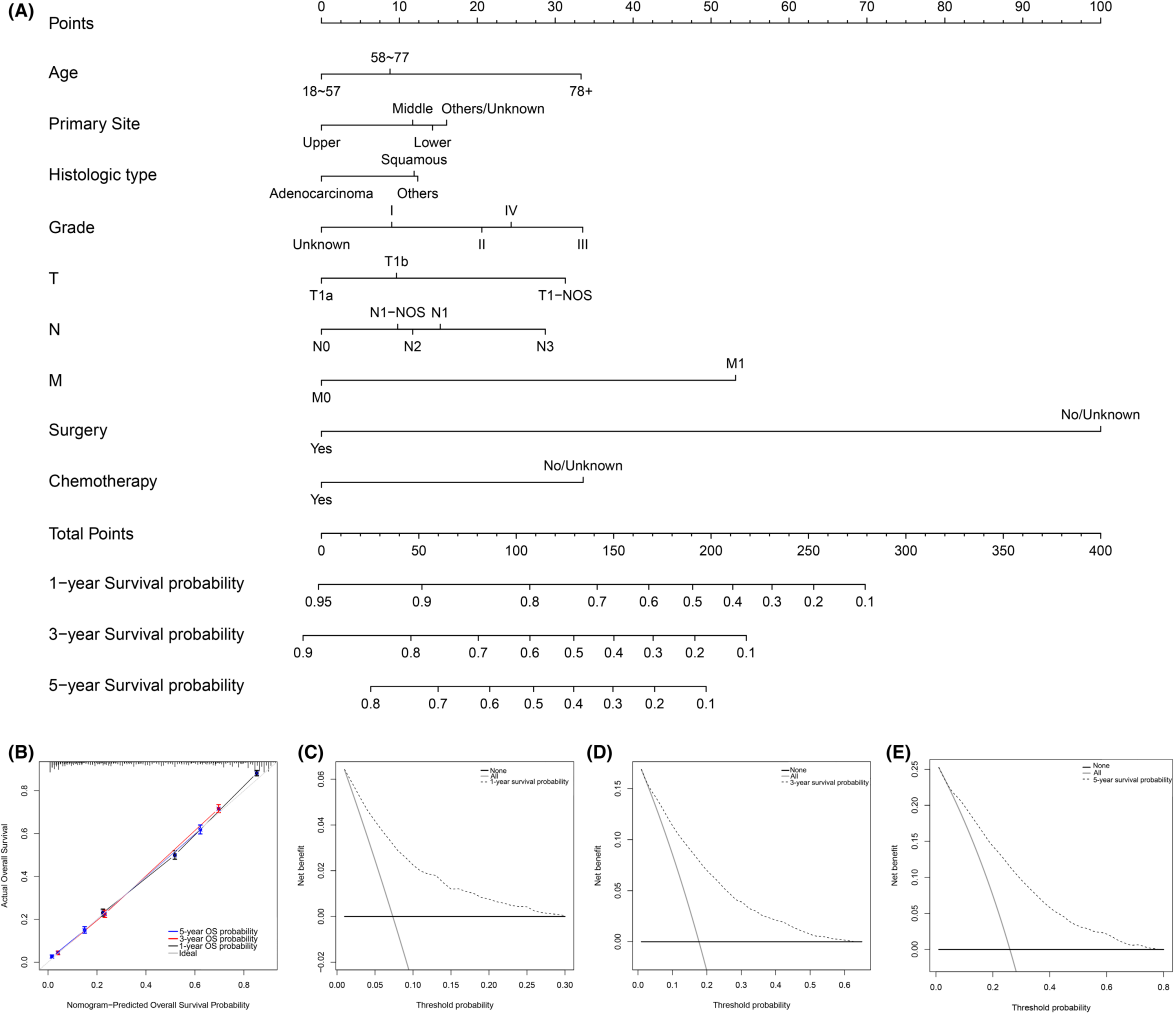
A prognostic nomogram (A) of the T1 EC population. Calibration curve (B), and DCA curves (C–E) for predicting 1‐, 3‐, and 5‐years survival of the population

Significant variables were selected to establish a nomogram for predicting OS probability of patients with T1 EC, as shown in Figure 4A. The concordance index was 0.752. Calibration curves (Figure 4B) for 1‐, 3‐, and 5‐year OS were close to the standard curve, which showed an advantageous predictive accuracy. Furthermore, as shown in the DCA curves (Figure 4C–E), the most beneficial threshold probability of the nomogram was 0–0.3, 0–0.6, and 0–0.8 for predicting 1‐, 3‐ and 5‐year OS probability, which demonstrated a broad range of clinical utility.

### 3.5 Clinical effects

**FIGURE 5:**
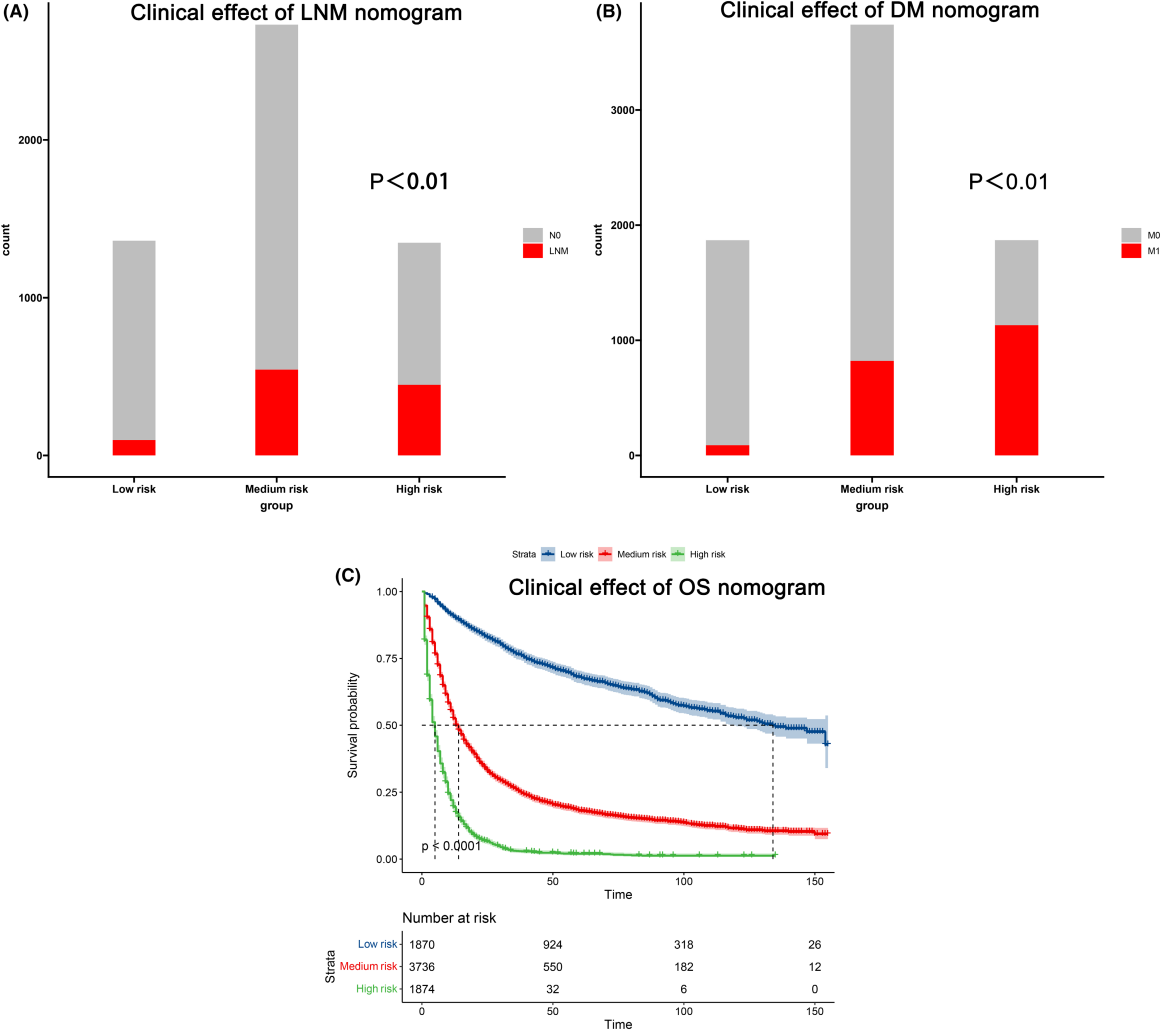
The occurrence of LNM (A) and DM (B) in different risk subgroups. Survival analysis of different risk subgroups of the OS nomogram

Cohort N was divided into three groups (low‐, medium‐, and high‐risk) according to the interquartile range of the LNM nomogram score. The corresponding scores for the three subgroups were 0–167, 168–269, and 270+, respectively. The occurrence of LNM was significantly different in these subgroups, as shown in Figure 5A. We divided cohort M into three subgroups similarly as follows: low‐risk group (0–135); medium‐risk group (136–226); high‐risk group (227+). The chi‐squared test shows a significant difference in the DM rate in these subgroups (*p* < 0.01) (Figure 5B). OS probability also showed statistically significant differences in the three subgroups (low‐risk group: 0–91, medium‐risk group: 92–231, and high‐risk group: 232+) (Figure 5C).

### 3.6 Survival analysis

**FIGURE 6:**
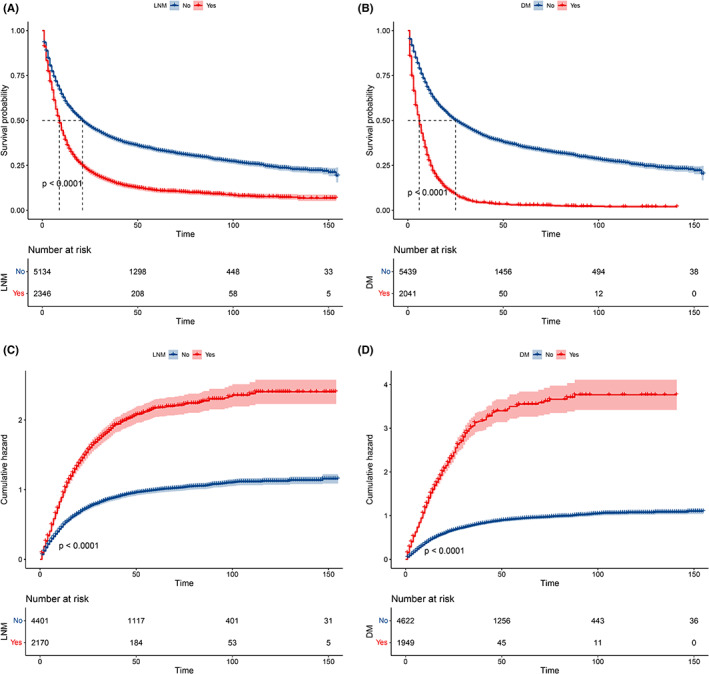
Survival analysis of LNM and DM on OS (A and B) and CSS (C and D) in T1 EC patients

KM and Gray methods were used to assess the influence of LNM and DM on OS in patients with T1 EC. As shown in Figure 6A,B, LNM (HR 1.90, 95% CI 1.8–2.01, *p* < 0.01) and DM (HR 3.32, 95% CI 3.13–3.51, *p* < 0.01) were significantly associated with OS. As for CSS, similar results were obtained for LNM (subdistribution hazard ratio [SHR] 2.01, 95% CI 1.9–2.14, *p* < 0.01) and DM (SHR 3.50, 95% CI 3.29–3.72, *p* < 0.01) in Figure 6C,D.

**FIGURE 7:**
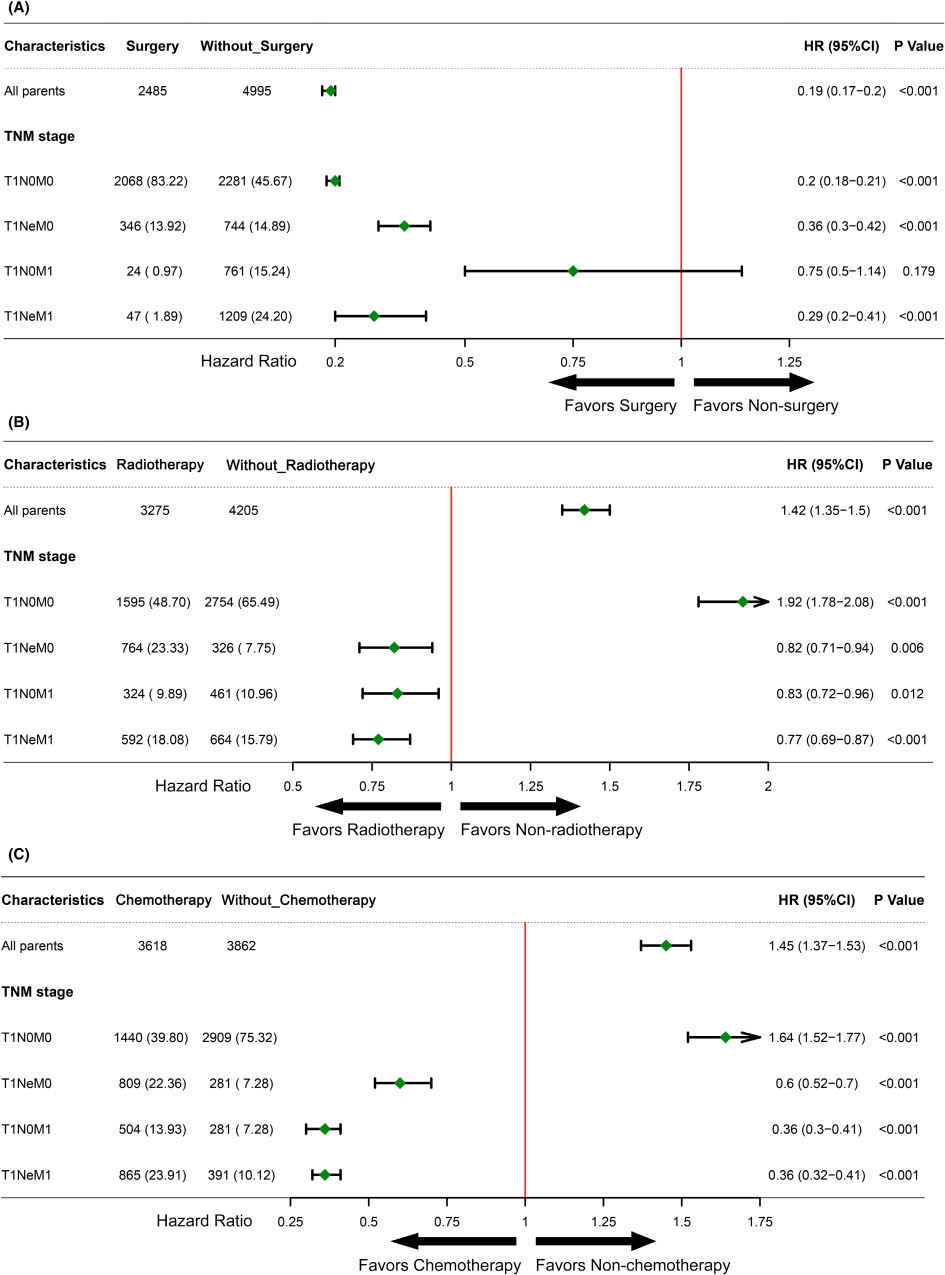
Forest plot of the association between different treatment modalities to the T1 esophageal cancer and overall survival by subgroup. Ne: number of LNM events

We explored the influence of various treatment modalities on the prognosis ofT1 EC patients with different lymph node and distant metastatic status, as shown in Figure 7. In patients with T1N0M0 EC, surgery (HR 0.2, 95% CI 0.18–0.21, *p* < 0.001) indicated a favorable prognosis, whereas radiotherapy (HR 1.92, 95% CI 1.78–2.08, *p* < 0.001) and chemotherapy (HR 1.64, 95% CI 1.52–1.77, *p* < 0.001) were associated with poor prognosis. As for patients with T1 EC and LNM or DM, radiotherapy and chemotherapy were associated with a survival benefit. Surgery offered significant survival benefits in patients with any N or M status except T1N0M1 EC (HR 0.75, 95% CI 0.5–1.14, *p* = 0.179).

## 4 DISCUSSION

Due to the heterogeneity of tumors and varying clinical features, patients with early EC can have different prognoses. Patients with early EC and different lymph node and DM status need individualized treatment. In terms of T1 EC without LNM or DM, endoscopic resection could be curative for mucosal (T1a) carcinoma with a strict indication. In the case of submucosal infiltration (<500 μm), endoscopic therapy could be an adequate alternative to surgery in esophageal adenocarcinoma; for T1 EC submucosal carcinoma, surgery is still the gold standard, with a higher recommendation.^2^ For T1 EC with LNM, however, which accounts for roughly 30% of cases,^6^ patients require radical resection and regional lymph node dissection. In terms of patients with early EC with metastatic disease who lose the opportunity for surgery, they need to receive comprehensive treatment, such as chemotherapy, radiation, targeted therapy, and immunotherapy. EUS plays a major role in the staging of early EC, with high accuracy (over 80%^12^) for the infiltration depth (T); however, it is limited in distinguishing regional LNM. EUS is also limited in its assessment of metastatic disease. PET‐CT is more sensitive, which could exclude 88% of distant metastases.^11^ However, it is not a procedure routinely used because it is not cost‐effective in the initial staging of early EC. Given the inaccuracies in the current staging system for identifying LNM and DM, clinicians cannot develop an appropriate treatment strategy for patients with early EC preoperatively. Establishing a tool for forecasting LNM and DM in patients with T1 EC would improve the selection of preoperative therapy. It is also of great value to evaluate prognosis based on clinical features in patients with early EC.

In this study, we established three clinical models to predict occult nodal metastases, distant metastases, and OS in patients with early EC based on the SEER program. We then internally validated nomograms by calibration curve, ROC, DCA, and CIC. We also analyzed the influence of various treatments on prognosis in the early EC population. The LNM nomogram incorporates six factors: age at diagnosis, marital status, race, histology, T stage, and histological differentiation. The DM nomogram involves six variables: age at diagnosis, primary site, histology, histological differentiation, T stage, and N stage. Nine factors were selected to build the OS nomogram: age at diagnosis, primary site, histology, grade, T stage, N stage, M stage, chemotherapy, and surgery.

The three nomograms described in our study illustrated a great degree of accuracy and discrimination. The AUC value of the LNM and DM model was 0.668 and 0.807, respectively, and the C‐index was 0.752 for the OS nomogram. Three clinical models demonstrated good clinical effects in inappropriate range of threshold probability. We divided patients into three subgroups according to the nomograms' interquartile scores. The stacked bar charts and KM curves were plotted to identify the discrimination of the nomograms. Finally, we performed a survival analysis based on stratification by N and M status.

In terms of the LNM nomogram, the T stage and histological differentiation accounted for the largest portion. It had previously been reported that the depth of tumor invasion (T) was related to LNM in early EC,^13^, ^14^ which was consistent with our findings. Submucosal EC has a higher rate of LNM, which could be explained by the abundant lymphatic drainage. Many reports previously confirmed a significant association between histologic differentiation and LNM in early EC; poorer differentiation was related to a higher LNM rate.^5^, ^15^ Compared with a well‐differentiated tumor, undifferentiated and poorly differentiated cancers had 2.17 and 2.77 (both *p* < 0.05) times the risk of LNM. It has been confirmed that younger age is an independent predictive factor for LNM in some solid cancers.^16^, ^17^ We found a significant relationship between age and LNM, which was consistent with previous studies. Interestingly, the risk of lymph node spread was higher in squamous cell EC compared with esophageal adenocarcinoma in our study, which could be explained by a poorer biological behavior in squamous cell cancer.

In the DM nomogram, T stage and N stage accounted for the largest portion. Not surprisingly, patients with LNM and deeper infiltration of tumors were more likely to acquire DM. Histologic differentiation is a manifestation of tumor invasiveness and malignancy. A higher grade often indicates a higher possibility of invasion to surrounding organs and tissues, and often to more distant sites, which was consistent with the results of our study. As for age, younger patients are more likely to have distant metastasis, which was similar to the results of the LNM nomogram. Previously reported studies have confirmed a strong relationship between younger age and LNM,^5^ which can lead to a higher risk of DM in T1 EC. Compared with tumors located at the upper esophagus and squamous cancer, adenocarcinoma located in the middle or lower had a higher risk of DM. We could not find such a phenomenon in the previously reported literature. We assumed that most adenocarcinomas of the esophagus are located in the lower part of the esophagus.^18^ Compared with esophageal squamous carcinoma, esophageal adenocarcinoma has a higher frequency of circulating tumor cells,^19^ which could explain the higher risk of DM in esophageal adenocarcinoma.

In the OS nomogram, surgery and M stage accounted for the largest part. Patients without surgery are expected to have a poorer prognosis in T1 EC. The stages (T, N, and M) are considered the most consistent prognostic factor in most of the solid tumors, and they remained significant predictors of OS in our study. A poorer histologic differentiation was related to a poorer prognosis due to the higher risk of LNM and DM, mentioned above. Age is an accurate reflection of the state of the body, and to a certain extent, reflects the patient's comprehensive status. Consistently, Ngamruengphong et al. reported that older age was a prognostic factor of survival in early EC patients.^20^ In terms of histologic type, we found that esophageal squamous carcinoma had a poorer prognosis compared with esophageal adenocarcinoma. However, few studies have explored this point adequately until now. A previous study had reported a survival benefit of esophageal adenocarcinoma compared with squamous cell cancer.^21^ However, others could not demonstrate a prognostic difference between adenocarcinoma and squamous cell carcinoma in early EC patients.^22^, ^23^ The reason for the association between histological type and prognosis remains unclear. Adenocarcinoma was associated with more favorable parameters compared with squamous cell cancer due to its heterogeneous biological behavior, such as a favorable growth pattern, better degree of histological differentiation, and less lymphatic vessel invasion. We hypothesized that their combination would contribute to a better prognosis of adenocarcinoma. More studies are needed in this respect in the future.

For the choice of treatment, the survival analysis demonstrated a survival benefit for surgery in all the patients with T1 EC compared with chemotherapy and radiotherapy. On subgroup analysis, for patients with LNM or DM, chemotherapy and radiotherapy showed a significant survival advantage. Although the use of endoscopic therapy in early EC has been continuously increasing over the last decade, primary surgery still shows an advantage in less LNM and better long‐term survival.^24^ Chemoradiotherapy remains highly controversial in patients with early EC, due to radiotherapy‐associated adverse effects and a relapse rate up to 30%.^25^ Some retrospective studies compared endoscopic therapy plus chemoradiation with other treatments and found acceptable toxicity with promising tumor control.^26^, ^27^ Further prospective research exploring the optimal candidates for this combination therapy, as well as the radiation dose, technology, and systemic treatment, are warranted in the future.

Studies reporting nomograms to predict LNM or survival in patients with early EC have been published previously. Duan et al.^28^ reported a model to predict LNM in early esophageal squamous cell carcinoma (ESCC). Three items (tumor size, tumor grade, and stage T) were included in the nomogram. Zheng et al.^29^ developed a nomogram including four factors (depth of infiltration, tumor size, tumor grade, and lymphovascular invasion) to predict LNM in early ESCC. The two nomograms mentioned above were validated externally using another case cohort in their cancer center and demonstrated a better discrimination ability. Their models included patients who underwent esophagectomy and were limited to squamous cell carcinoma. The difference in the discrimination ability could be due to more histopathology parameters and better data quality. Our nomograms incorporated the entire population from the SEER database and could apply to preoperative patients. Few studies have reported nomograms to predict survival in early EC. Jia et al.^30^ recently developed a prognostic nomogram for patients with early EC based on the SEER program. Their study explored the impact of various treatment options on the prognosis of patients with T1 EC. They subdivided the treatment options and found that endoscopic therapy was not inferior to esophagectomy. In the current study, the subgroup analysis showed a survival benefit for patients with early EC undergoing surgery, which deserves to be validated in a prospective study.

Some limitations in our research merit discussion. First, a retrospective dataset from the SEER program was used to identify T1 EC patients for inclusion in the study; patients might not be well chosen by this model if they did not match the typical demographics. Additionally, several important factors were not documented in the SEER database, including chemoradiotherapy protocols, surgical methods, and information on treatment after recurrences. Last but not least, our study has not been externally validated. We have collected all the data of patients with T1 esophageal cancer who underwent esophagus resection at our medical center. There were less than 100 cases in our medical center, and none of them occurred LNM or DM, it needs to be further validated in a multi‐center, large sample cohort. However, we did use a representative data set to establish our models. Furthermore, we used the bootstrapping method to validate our predictive models internally, which has been proven to provide minimal error estimates.^31^

The deficiencies mentioned above, however, are balanced by the utility and strength of our research. Based on a large sample size of over 7000 patients, our models expand on models previously reported to predict LNM, DM, and prognosis in early EC. Unknown variables for stage T, stage N, and grade were included in our models to allow wide applicability of our nomograms. This simple, practical tool can provide additional information when the clinical stage is sometimes considered to be inaccurate. Moreover, our nomograms might facilitate and improve the selection of treatment, to improve the care for early EC patients.

## AUTHOR CONTRIBUTIONS

**Hong Chen:** Conceptualization (lead); resources (lead); software (lead); supervision (equal); validation (equal); visualization (equal); writing – original draft (lead); writing – review and editing (lead). **Junxian Wu:** Conceptualization (equal); resources (equal); software (equal). **Wanting Guo:** Conceptualization (equal); data curation (equal); validation (equal). **Lihang Yang:** Data curation (equal); formal analysis (equal). **Linbin Lu:** Resources (equal); software (equal); writing – review and editing (equal). **Yihong Lin:** Investigation (equal); methodology (equal). **Xuewen Wang:** Software (equal). **Yan Zhang:** Data curation (equal); resources (equal); supervision (equal); writing – review and editing (equal). **Xi Chen:** Data curation (equal); funding acquisition (equal); resources (equal); supervision (equal); writing – review and editing (equal).

## FUNDING INFORMATION

This research received financial support from the Natural Science Foundation of Fujian Province (Nos 2018 J01352).

## CONFLICT OF INTEREST

The authors have no conflicts of interest to declare that are relevant to the content of this article.

## ETHICS APPROVAL AND PATIENTS INFORMED CONSENT

The IRB has confirmed that no ethical approval is required due to the anonymized, de‐identified data from the SEER database.

## Data Availability

The data and materials are available on reasonable request from the corresponding author.
